# Supplementary material for: Two level logistic regression analysis of factors influencing skilled birth attendant during delivery among Bangladeshi mothers: A nationally representative sample
Source: PLoS One. 2023 Sep 26;18(9):e0291790. doi: 10.1371/journal.pone.0291790 (PMC10522025; doi:10.1371/journal.pone.0291790)
Supplement: Attachment — Submitted filename: Response to Reviewers.docx [file pone.0291790.s001.docx]

**Date: May 24, 2023**

**Response to reviewers**

**Paper Title:** Two level logistic regression analysis of factors influencing skilled birth attendant during delivery among Bangladeshi mothers:  A nationally representative sample

**Journal Name:** PLOS ONE

**Manuscript ID:** PONE-D-22-27776

Dear Editor

Thank you very much for providing us valuable comments on our manuscript. We have modified and revised the manuscript accordingly; detailed point-by-point corrections are given below:

**Journal Requirements:**

When submitting your revision, we need you to address these additional requirements.

1. Please ensure that your manuscript meets PLOS ONE's style requirements, including those for file naming. The PLOS ONE style templates can be found at

https://journals.plos.org/plosone/s/file?id=wjVg/PLOSOne_formatting_sample_main_body.pdf and

<https://journals.plos.org/plosone/s/file?id=ba62/PLOSOne_formatting_sample_title_authors_affiliations.pdf>

**Response:** Thank you very much for providing Journal requirements. We have checked our manuscript, and we tried to follow PLOS ONE's style throughout the manuscript.

2. Thank you for stating the following financial disclosure:

 “The funders had no role in study design, data collection and analysis, decision to publish, or preparation of the manuscript.”

**Response:** Thank you.

At this time, please address the following queries:

a) Please clarify the sources of funding (financial or material support) for your study. List the grants or organizations that supported your study, including funding received from your institution.

b) State what role the funders took in the study. If the funders had no role in your study, please state: “The funders had no role in study design, data collection and analysis, decision to publish, or preparation of the manuscript.”

c) If any authors received a salary from any of your funders, please state which authors and which funders.

d) If you did not receive any funding for this study, please state: “The authors received no specific funding for this work.”

**Response:** Since we did not receive any funding for this study, we have stated “The authors received no specific funding for this work.”

Please include your amended statements within your cover letter; we will change the online submission form on your behalf.
3. Please review your reference list to ensure that it is complete and correct. If you have cited papers that have been retracted, please include the rationale for doing so in the manuscript text, or remove these references and replace them with relevant current references. Any changes to the reference list should be mentioned in the rebuttal letter that accompanies your revised manuscript. If you need to cite a retracted article, indicate the article’s retracted status in the References list and also include a citation and full reference for the retraction notice.
**Response:** We have checked, and the reference list and citations are ok.

**Reviewers' comments to the Author**

1. Is the manuscript technically sound, and do the data support the conclusions?

The manuscript must describe a technically sound piece of scientific research with data that supports the conclusions. Experiments must have been conducted rigorously, with appropriate controls, replication, and sample sizes. The conclusions must be drawn appropriately based on the data presented.

Reviewer #1: Yes

2. Has the statistical analysis been performed appropriately and rigorously?

Reviewer #1: Yes

3. Have the authors made all data underlying the findings in their manuscript fully available?

The [PLOS Data policy](http://www.plosone.org/static/policies.action#sharing) requires authors to make all data underlying the findings described in their manuscript fully available without restriction, with rare exception (please refer to the Data Availability Statement in the manuscript PDF file). The data should be provided as part of the manuscript or its supporting information, or deposited to a public repository. For example, in addition to summary statistics, the data points behind means, medians and variance measures should be available. If there are restrictions on publicly sharing data—e.g. participant privacy or use of data from a third party—those must be specified.

Reviewer #1: Yes

4. Is the manuscript presented in an intelligible fashion and written in standard English?

PLOS ONE does not copyedit accepted manuscripts, so the language in submitted articles must be clear, correct, and unambiguous. Any typographical or grammatical errors should be corrected at revision, so please note any specific errors here.

Reviewer #1: Yes

**Response:** Thank you very much for your comments on our manuscript.

**5. Review Comments to the Author**
Please use the space provided to explain your answers to the questions above. You may also include additional comments for the author, including concerns about dual publication, research ethics, or publication ethics. (Please upload your review as an attachment if it exceeds 20,000 characters)

**Reviewer #1: Comments to Authors**
1. I strongly encourage that instead of chi-square values, please consider presenting an estimate of unadjusted relative risk (or odds) and confidence intervals. This will enable separation of the factors into those associated with an increase in risk for skilled birth attendant during delivery and those associated with a decreased risk.

**Response:** We appreciate reviewer’s good comments. As reviewer’s comments, we have used both univariate (unadjusted) and multivariate (adjusted) binary logistic models to find the effect of socio-economic, demographic and other selected variables on skilled birth attendant. All variables with a p-value of <0.20 from the likelihood ratio test in the univariate model were included in the multivariate logistic regression model. We estimated unadjusted odds risk with its 95% confidence intervals instead of Chi-square test. Please see in Table 2.

2. In this study the two level logistic model was used to analysis the factors influencing the skilled birth attendant during delivery but ICC (intra class correlation) values not present in the report. Thus, please include ICC report to identify the total variation in skilled birth attendant during delivery due to regions.

**Response:** We appreciate reviewer’s important comment. Reviewer is right, the intra class correlation (ICC) should use to check whether multiple level model is necessary or not for hierarchical data. We know, Larsen and Merlo (2005) suggest using the median odds ratio (MOR) for dichotomies outcome instead of ICC but still we know some researchers use ICC. We think ICC or MOR is suitable for checking whether multiple level model is necessary or not, and MOR has been used in many publications such as (i) Islam M.A, Mamun A, Hossain M.M, Bharati P, Saw A, Lestrel PE, et al. (2019) Prevalence and factors associated with early initiation of breastfeeding among Bangladeshi mothers: A nationwide cross-sectional study. PLoS ONE 14(4): e0215733. https://doi.org/10.1371/ journal.pone.0215733.

In our present study, we have used MOR, we have mentioned the formula with the properties of MOR, and the value of MOR has been mentioned in page: 11; Line: 5-12.

We would like to thank the reviewers for the valuable comments. We have revised the documents to the best of our ability, but we will definitely be happy to provide further improvement if there are further clarifications required.

With best regards

Dr. Md. Golam Hossain

Professor of Health Research Group

Department of Statistics, University of Rajshahi

Rajshahi-6205, Bangladesh

E-mail: hossain95@yahoo.com
